# The influence of kinesio taping on trunk and lower extremity motions during different landing tasks: implications for anterior cruciate ligament injury

**DOI:** 10.1186/s40634-021-00339-w

**Published:** 2021-04-02

**Authors:** Bahram Sheikhi, Amir Letafatkar, Jennifer Hogg, Esmaiel Naseri-Mobaraki

**Affiliations:** 1 Faculty of Physical Education and Sports Sciences, Kharazmi University, Tehran, Iran; 2 Department of Biomechanics and Sports Injury, Faculty of Physical Education and Sports Sciences, Kharazmi University, Tehran, Iran; 3 Health & Human Performance Department, Graduate Athletic Training Program, University of Tennessee Chattanooga, Chattanooga, USA; 4 Faculty of Physical Education and Sports Sciences, University of Tehran, Tehran, Iran

**Keywords:** Kinesio tape, Anterior cruciate ligament, Knee, Landing, Joint kinematics

## Abstract

**Purpose:**

The purpose of the study was to investigate the influence of a 72-h KT application on trunk and lower extremity kinematics during different landing tasks.

**Methods:**

Twenty-nine competitive male athletes participated in this study. The sum of knee valgus and lateral trunk lean, symmetry index (SI), and peak angles of lateral trunk lean, hip flexion, knee abduction and flexion were assessed for all participants during single-leg drop landing (SLDL), single-leg vertical drop jump (SLVDJ), vertical drop jump (DLVDJ), and double leg forward jump (DLFJ), at baseline and seventy-two hours following KT application.

**Results:**

The KT application resulted in more knee flexion and abduction, sum of knee valgus and lateral trunk lean as compared with the non-KT condition during SLDL (P < 0.05). Nonetheless, there were no differences in SI, maximum angle of the lateral trunk lean during SLDL, SLVDJ, nor hip flexion, knee abduction, and flexion during DLVDJ, and DLFJ tasks (P > 0.05).

**Conclusions:**

The research findings suggest that KT after 72-h application may improve knee abduction and sum of knee valgus and lateral trunk lean during SLDL, knee flexion during SLDL and SLVDJ in individuals displaying risky single-leg kinematics. Therefore, KT application may marginally improve high-risk landing kinematics in competitive male athletes.

**Level of evidence:**

Level III.

## Introduction

Anterior cruciate ligament (ACL) injuries frequently occur in non-contact situations such as landing [12, 22]. In order to assess ACL injury risk and develop potential preventive strategies, researchers have typically measured biomechanical characteristics during various landing tasks [17].

Poor sagittal and frontal plane movement patterns are believed to increase knee injury risk in athletes [10, 25]. Specifically, dynamic malalignment patterns comprised of greater ipsilateral trunk lean, hip adduction, hip internal rotation, knee valgus (KV) and tibial internal or external rotation, in addition to less hip and knee flexion, have been associated with greater knee joint loading and subsequently higher non-contact ACL injury risk during landing tasks. Characterized by an erect landing posture and less sagittal plane trunk displacement, stiff landings result in greater ground reaction forces [12], external knee abduction and flexion moments, and smaller external hip flexion moments [8, 10]. In the sagittal plane, the trunk and lower extremity work in a coupled fashion to attenuate landing forces, such that greater motion at one joint is typically accompanied by corresponding motion at adjacent joints, allowing for improved force absorption [9, 12]. In the frontal plane, greater KV displacement is a primary predictor of non-contact ACL injury risk [12]. A combination of increased two-dimensional (2D) measured KV and ipsilateral trunk lean was associated with increased external peak knee abduction moment during a single leg vertical drop jump (SLVDJ). Increased lateral trunk lean causes the ground reaction vector to pass lateral to the knee joint, thereby creating an external knee abduction moment. Greater KV allows the ground reaction force (GRF) to exert even greater frontal plane torque upon the knee joint [8, 10]. In addition to intra-limb kinematics, inter-limb asymmetries are also shown to increase the occurrence of sport-related injuries. Reduced asymmetry, specifically in regards to knee flexion, knee abduction, and hip flexion, can prevent lower extremity injury [38].

While it is accepted that dynamic trunk, hip, and knee alignment influence one’s risk for ACL injury [4, 9, 40], it is largely unknown if kinesio taping (KT) may improve high-risk kinematics. KT is an elastic therapeutic tape used to prevent and treat sports injuries and various musculoskeletal conditions [21, 43]. KT has several positive effects: improving lymphatic flow by increasing interstitial space, supporting muscles and joints, and correcting articular malalignment and function [21, 28]. Moreover, KT is known for improving function, stability, proprioception [16], and force production of the muscle [21, 35]. Also, through tactile input, KT has been able to stimulate cutaneous mechanoreceptors and alter motoneurons [6].

According to the author's knowledge, no study has tried to examine the sum of knee valgus and lateral trunk lean, lateral trunk lean, knee abduction, hip and knee flexion, and asymmetry of landing tasks after KT application. Using methods such as KT may help correct dynamic malalignment patterns, and reduce knee injury risk [5, 30, 32].

Investigating different landing tasks can enhance our understanding of the influence of KT on motion and injury risk. Accordingly, this study’s aim was to investigate the influence of KT on trunk, hip and knee motions during a single-leg drop landing (SLDL), SLVDJ, double leg vertical drop jump (DLVDJ), and double leg forward jump (DLFJ) tasks. The primary hypothesis was that KT would improve peak sagittal and frontal plane angles of the trunk, hip and knee during SLDL, SLVDJ, DLVDJ and DLFJ.

## Materials and methods

### Study design and participants

**Fig. 1:**
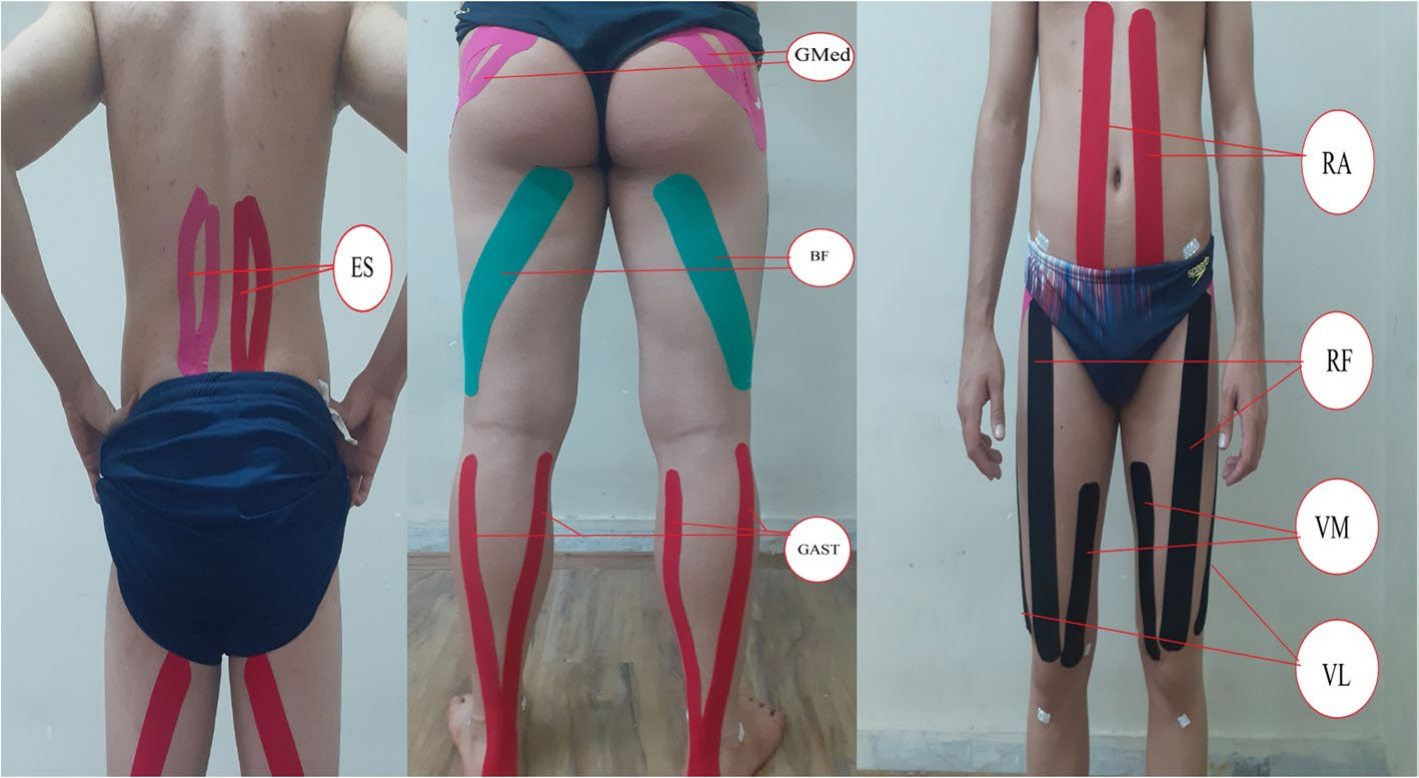
Kinesio taping applications: gastrocnemius (GAST), biceps femoris (BF), vastus lateralis (VL), vastus medialis (VM), rectus femoris (RF), gluteus medius (GMed), rectus abdominis (RA), and erector spinae (ES) muscles

A pretest–posttest design was used in the current study. Trunk, hip and knee kinematics were assessed during SLDL, SLVDJ, DLVDJ and DLFJ. Following baseline testing, KT was applied to gastrocnemius, biceps femoris, semitendinosus, semimembranosus, vastus lateralis, vastus medialis, rectus femoris, gluteus medius, rectus abdominis, and erector spinae muscles (Fig. 1). Each participant returned for follow-up testing 72 h later.

Twenty-nine competitive males (mean ± SD, 23.2 ± 2.1 years; 185.8 ± 7.0 cm; 77.0 ± 7.3 kg; KV = 13.1 ± 1.9 degrees) were included in this study. Only male participants were included to avoid sex-specific differences in neuromuscular performance [31]. Six participants were right limb dominant and 23 were left limb dominant, as determined by asking participants which leg they preferred to land on following a jump. Participants had an average of 8.4 ± 2.1 years of experience in their respective jumping and multidirectional sports (8 soccer athletes, 10 basketball athletes, 5 handball athletes, and 6 volleyball athletes). Prior to data collection, this study had been approved by the research ethics committee of the faculty of physical education and sport science of the Tarbiat Modarres University. The study was performed in accordance with the ethical standards in the World Medical Association Declaration of Helsinki (2002). Ethical approval was obtained from the Ethical Committee of the Medical Faculty of the Tarbiat Modarres and the ethical standards in sport and exercise science research were respected.

To be eligible to participate, each participant was required to meet the following criteria: 18–26 years of age, no history of surgery in the lower extremity in the previous six months, no history of non-corrected neurological, vestibular, visual and/or hearing impairments, no musculoskeletal injury that could interfere with or contraindicate the assessment procedures [7, 40], and must have had no allergy to adhesive material [5] nor any other conditions that prevent them from participating at the maximal effort in sporting activities [4], Additionally, KV of more than 10 degrees during a single leg squat test (SLS) was required to participate in this study. The SLS test was used in this study based on the protocol introduced by Ugalde et al. [41]. One hundred-three athletes were assessed for eligibility; which 74 athletes did not meet inclusion criteria.

### Procedures

#### KT application

Kinesio tape (Kinesio Tex Gold™, FP, 5 cm wide) was used in this study according to the technique described by Kase et al. [21], and was applied with 50%-75% tape tension. KT was applied to muscles (gastrocnemius, biceps femoris, vastus lateralis, vastus medialis, rectus femoris, gluteus medius, rectus abdominis, and erector spinae) that show the greatest impact on trunk, hip and knee motion during landing (Fig. 1, Table 1). In order to standardize tension during the KT application, the distances between the origin and insertion of all participants’ muscles were measured. Prior to the adhesive KT application, the skin was first cleaned at the site of application using an alcohol 70 GL prep pad, and excess hair was trimmed so as to ensure KT adherence. Furthermore, the participants’ skin sensitivity was tested with a KT test patch over a period of 24 h prior to the study. All strips were bilaterally applied to the trunk and lower extremity muscles by the same trained researcher.

**Table 1:** KT application was applied in the following order

Muscles	KT application
Gastrocnemius	The KT was split into a Y-strip so that each side could be longitudinally taped along with origin of the medial and lateral gastrocnemius muscles. Both the proximal ends of the Y-strip were placed, without tension, 4 cm below the popliteal line with the ankle in the neutral position. The proximal half of the strip was then stretched and placed on the calf up to the marked midpoint with the participant’s ankle at maximum dorsiflexion. The distal half of the strip was also stretched and placed from the midpoint to the upper part of the calcaneus posterior tuberosity with the participants ankle still at maximum dorsiflexion, and distal end of the Y-strip was then placed, without tension, with the ankle back in neutral position [19]
Biceps Femoris	As regards biceps femoris KT, the participant was positioned side lying with the knee in extension, the hip in flexion, the hip medially rotated, and the contralateral leg slightly bent for stability. KT was applied from the ischial tuberosity to the posterior region of the fibular head [31]
Quadriceps	KT was applied on quadriceps muscle, from the proximal to the distal [12]. Also, it was applied to the RF from 10 cm below the anterior superior iliac spine (ASIS) to the upper edge of the patella [20]. The strip was fixed on the VL muscle from the greater trochanter to the lateral patella edge. For the VM muscle, KT was applied to the middle third from the medial region of the thigh to the medial patella edge. This application was performed with participants standing on one foot, with the hip of the dominant limb at 0° and knee flexed at 90° [17]. The individuals were requested to perform a maximal extension of their knee in order to obtain length measurements, and to make KT final adjustments prior to its application
Gluteus Medius	For the gluteus medius, KT was applied from iliac crest to GT in side lying position. Participants were asked to take the side-lying position with 90° hip flexion, adduction and internal rotation. Y strip was used from insertion to origin. Base of the Y strip was applied on the lateral surface of the GT with no tension. Anterior tail was applied towards the ASIS with light or paper off tension and the last 1–2 inches with no tension. Posterior tail was applied towards PSIS with a similar tension mentioned above [12, 16]
Erector Spinae	The tape was bilaterally placed over the erector spinae muscles, parallel to the spinous processes of the lumbar vertebrae [44], starting near the posterior superior iliac crest [45]
Rectus Abdominis	Two pieces of tape were longitudinally applied on the rectus abdominis from the level of the xiphoid process to the pubic symphysis level [46]

#### Landing tasks

Prior to the tests, the participants executed a standardized warm-up protocol consisting of a series of double-leg squats (2 × 8 repetitions) and double-leg maximum jumps (2 × 5 repetitions), followed by calf-stretching with a straight and bent knee [37]. Ten minutes were allotted to each participant to perform self-directed stretches and warm-ups. Furthermore, the participants were allowed to familiarize themselves with the test procedures by performing two or three practice repetitions before each test.

The participants completed four tests: SLDL, SLVDJ, DLVDJ and DLFJ. A single tester provided the participants with all the instructions regarding all four tests. Both legs were tested in the single-leg tests. The tests were also randomly ordered. All participants completed three practice trials and three successful test trials of each task. At least 60 s’ rest was given between each repetition and two minutes’ rest after each task to minimize fatigue. All measurements were conducted in the university biomechanics laboratory.

#### Single-Leg Drop Landing (SLDL)

Participants performed SLDL from a 30-cm box [42]. After landing, this position was maintained for five seconds. A trial was not deemed valid if the other leg touched the ground or if the participants was clearly out of balance or fell during the test.

#### Single-Leg Vertical Drop Jump (SLVDJ)

Each participant performed a SLVDJ on his dominant leg. A SLVDJ consisted of dropping from a 10-cm box, landing on one limb, completing an immediate maximal vertical jump, and landing again [37]. A trial was deemed invalid if the participant jumped off the box instead of just dropping, if the other leg touched the ground or if the participant was clearly out of balance or fell during the test.

#### Double Leg Vertical Drop Jump (DLVDJ)

As for the DLVDJ, the participants were instructed to drop from a 30-cm height box, land on both limbs, and immediately perform a maximal vertical jump [19, 37, 39]. A trial was not considered valid if the participant lost balance or fell during the performance.

#### Double Leg Forward Jump (DLFJ)

For the DLFJ, a 30-cm box was placed at a distance equal to 50% of the participant’s height from the front edge of the force plates. From this distance, the participant jumped onto a set of plates with both feet simultaneously, and subsequently performed a maximum vertical jump [4]. A trial was not deemed valid if the participant lost balance or fell during the performance.

### Data Reduction

#### Two-dimensional video analysis

Different landing tasks were captured with two standard digital video cameras (Sony HDR-PJ675). The video cameras were placed on tripods perpendicular to the sagittal and frontal planes, at a height of 0.6 m and a distance of 3.5 m from the force plates. Also, markers were bilaterally placed on the acromioclavicular (AC) joint, manubrium sterni, anterior superior iliac spine (ASIS), greater trochanter (GT), medial and lateral femoral epicondyles, and medial and lateral malleolus [10, 18].

In the frontal plane, lateral trunk lean was defined as the angle formed by vertical and a line from the ipsilateral ASIS to the manubrium sterni. Knee abduction angle was delimited as the angle formed by a segmented line from the ASIS to the knee joint center to the ankle joint center. In the sagittal plane, knee flexion angle was defined as the angle formed by a segmented line from the GT to the lateral femoral epicondyle to the lateral malleolus. Hip flexion angle was defined as the angle formed by a segmented line from the lateral femoral epicondyle to the GT to the AC joint [9]. The video recordings were analyzed using the Kinovea software (version 0.8.15). The ankle joint center was defined as the mid-point of the lateral and medial malleolus markers, and the knee joint center was described as the mid-point of the lateral and medial femoral epicondyle markers [1]. Joint angles were averaged across the three trials and used for statistical analysis. The point of maximum knee flexion during landing tasks was visually determined in Kinovea and was defined as the time point where no downward or upward movement occurred at the knee. At the point of maximal knee flexion, a digital picture of each trial was taken. All angles were drawn on the same digital picture and measured by a single investigator blinded to testing session.

#### Symmetry Index (SI)

As regards SLDL and SLVDJ, the degree of asymmetry between the dominant and non-dominant limb was then computed using the SI (Eq. 1), [38].

1 $$\mathrm{Symmetry}\;\mathrm{Index}=\frac{2(\mathrm{dominant}\;\mathrm{limb}\;-\;\mathrm{non}\;\mathrm{dominant}\;\mathrm{limb})}{(\mathrm{dominant}\;\mathrm{limb}\;+\;\mathrm{non}\;\mathrm{dominant}\;\mathrm{limb})}\times100\%$$

### Statistical analyses

An a priori power analysis was performed using G*Power software (version 3.1.9.2, written by Franz Faul, university Kiel, Germany). Given a medium effect size of 0.25, alpha level of 0.05, and a power of 0.8, a minimum of 24 participants was needed for this study.

Normality of variables were assessed using the Shapiro Wilk Test. Paired t-tests were performed to determine the influence of KT on the sum of knee valgus and lateral trunk lean, SI, and maximum angles of lateral trunk lean, hip flexion, knee abduction and knee flexion during the different landing tasks. Furthermore, magnitudes of the differences were examined using Cohen’s *d* effect size (ES), and were interpreted as follows: < 0.35—trivial; 0.35–0.8—small; 0.8–1.5—moderate; > 1.5 – large [3]. Change percentages were also calculated to compare follow-up testing with baseline testing.

Statistical significance was set a priori at ≤ 0.05. Data were analyzed using SPSS (version 16.0; SPSS Inc., Chicago, IL).

## Results

### Knee abduction

For knee abduction, within-subject differences for SLDL in the dominant leg (ES (95%CI) = 0.65 (0.24 to 1.05); *P* = 0.022) and non-dominant leg (ES (95%CI) = 0.61 (0.21 to 1.00); *P* = 0.014) were significant. SLVDJ (dominant leg: ES (95%CI) = -0.42 (-0.80 to -0.04); *P* = 0.083; non-dominant leg: ES (95%CI) = 0.14 (-0.23 to 0.50); *P* = 0.678), DLVDJ (ES (95%CI) = 0.03 (-0.34 to 0.39); *P* = 0.926) and DLFJ (ES (95%CI) = -0.25 (-0.62 to 0.12); *P* = 0.204), differences were not significant (Tables 2 and 3).

**Table 2:** Within-group changes in peak angles of knee abduction, knee flexion, hip flexion, lateral trunk lean, sum of knee valgus and lateral trunk lean during SLDL, SLVDJ tasks

**Landing Characteristic (°)**	**Task**	**Landing Leg**	**Without KT (Baseline)**^α^	**With KT**^α^	***P value***	**Effect size**^**†**^** and 95% Confidence Interval** (Lower limit -Upper limit)	**Change Relative to Baseline**^**‡**^** (%)**
**Knee abduction**	SLDL	Dominant	169.0 ± 6.3	172.0 ± 4.6	0.022*	0.65 (0.24 to 1.05)	↑ 1.8
		Non-dominant	168.3 ± 4.8	171.7 ± 5.6	0.014*	0.61 (0.21 to 1.00)	↑ 2.0
	SLVDJ	Dominant	169.0 ± 5.7	166.4 ± 6.3	0.083	-0.42 (-0.80 to -0.04)	↓ 1.6
		Non-dominant	167.5 ± 8.2	168.3 ± 6.2	0.678	0.14 (-0.23 to 0.50)	↑ 0.5
**Knee flexion**	SLDL	Dominant	103.3 ± 7.1	98.8 ± 9.9	0.018*	-0.45 (-0.83 to -0.07)	↓ 4.3
		Non-dominant	96.4 ± 8.9	91.6 ± 10.5	0.031*	-0.46 (-0.84 to -0.07)	↓ 5.0
	SLVDJ	Dominant	114.2 ± 9.0	108.6 ± 9.7	0.034*	-0.57 (-0.96 to -0.18)	↓ 4.9
		Non-dominant	112.9 ± 8.4	109.6 ± 7.2	0.040*	-0.45 (-0.83 to -0.07)	↓ 2.9
**Hip flexion**	SLDL	Dominant	87.4 ± 13.2	80.5 ± 12.9	0.060	-0.53 (-0.91 to -0.14)	↓ 7.8
		Non-dominant	94.6 ± 21.9	91.5 ± 9.2	0.553	-0.34 (-0.71 to 0.04)	↓ 3.3
	SLVDJ	Dominant	124.9 ± 14.2	119.9 ± 6.1	0.098	-0.82 (-1.24 to -0.40)	↓ 4.0
		Non-dominant	132.5 ± 11.3	125.9 ± 21.3	0.152	-0.31 (-0.68 to 0.07)	↓ 4.9
**Lateral trunk lean**	SLDL	Dominant	9.1 ± 5.1	9.6 ± 6.7	0.619	0.08 (-0.29 to 0.44)	↑ 5.6
		Non-dominant	11.1 ± 6.1	11.2 ± 8.0	0.924	0.01 (-0.35 to 0.38)	↑ 1.0
	SLVDJ	Dominant	8.3 ± 5.4	8.4 ± 5.5	0.850	0.02 (-0.34 to 0.38)	↑ 1.3
		Non-dominant	7.9 ± 4.8	6.6 ± 4.4	0.199	-0.29 (-0.66 to 0.09)	↓16.1
**Sum of knee valgus and lateral trunk lean**	SLDL	Dominant	178.1 ± 7.8	181.6 ± 9.4	0.033*	0.37 (-0.004 to 0.75)	↑ 2.0
		Non-dominant	179.3 ± 8.2	182.9 ± 11.6	0.044*	0.31 (-0.07 to 0.68)	↑ 2.0
	SLVDJ	Dominant	177.3 ± 6.9	174.8 ± 7.9	0.097	-0.32 (-0.69 to 0.05)	↓ 1.4
		Non-dominant	175.3 ± 8.9	174.9 ± 7.5	0.860	-0.05 (-0.41 to 0.31)	↓ 0.2

Abbreviation:^α^, Values stands for Mean ± SD; *SLDL* Single-leg drop landing, *SLVDJ* Single-leg vertical drop jump, *KT* Kinesio taping; †, Cohen’s d; *_,_ significant difference (*P* < .05); ‡, percent change relative to baseline (↓decrease, ↑ increase)

**Table 3:** Peak angles of knee abduction, knee flexion and hip flexion during DLVDJ, DLFJ tasks

**Landing Characteristic (°)**	**Task**	**Without KT (Baseline)**^α^	**With KT**^α^	***P value***	**Effect size**^**†**^** and 95% Confidence Interval** (Lower limit -Upper limit)	**Change Relative to Baseline**^**‡**^** (%)**
**Knee abduction**	DLVDJ	169.4 ± 6.3	169.5 ± 4.8	0.926	0.03(-0.34 to 0.39)	↑ 0.1
	DLFJ	168.5 ± 5.0	166.1 ± 9.6	0.204	-0.25 (-0.62 to 0.12)	↓ 1.4
**Knee flexion**	DLVDJ	94.3 ± 9.2	93.3 ± 11.0	0.690	-0.08 (-0.45 to 0.28)	↓ 1.0
	DLFJ	96.7 ± 11.9	94.8 ± 12.5	0.550	-0.15 (-0.51 to 0.21)	↓ 2.0
**Hip flexion**	DLVDJ	101.3 ± 22.2	106.0 ± 24.1	0.481	0.20 (-0.17 to 0.56)	↑ 4.7
	DLFJ	101.7 ± 21.1	95.2 ± 13.2	0.128	-0.49 (-0.87 to -0.10)	↓ 6.4

Abbreviation:^α^, Values stands for Mean ± SD; *DLVDJ* Double leg vertical drop jump, *DLFJ* Double leg forward jump, *KT* Kinesio taping; †, Cohen’s d; ‡, percent change relative to baseline (↓decrease, ↑ increase)

### Knee flexion

During SLDL and SLVDJ, the KT application resulted in more knee flexion as compared to the non-KT condition. The within-subject difference was significant for SLDL (dominant leg: ES (95%CI) = -0.45 (-0.83 to -0.07); *P* = 0.018; non-dominant leg: ES (95%CI) = -0.46 (-0.84 to -0.07); *P* = 0.031) and SLVDJ (dominant leg: ES (95%CI) = -0.57 (-0.96 to -0.18); *P* = 0.034; non-dominant leg: ES (95%CI) = -0.45(-0.83 to -0.07); *P* = 0.040). Conversely, in the DLVDJ (ES (95%CI) = -0.08 (-0.45 to 0.28); *P* = 0.690) and DLFJ (ES (95%CI) = -0.15 (-0.51 to 0.21); *P* = 0.550), significant differences were not observed (Tables 2 and 3).

### Hip flexion

For hip flexion, within-subject differences were not significant for SLDL (dominant leg; ES (95%CI) = -0.53 (-0.91 to -0.14); *P* = 0.060, and non-dominant leg; ES (95%CI) = 0.34 (-0.71 to 0.04); *P* = 0.553), SLVDJ (dominant leg; ES (95%CI) = -0.82 (-1.24 to -0.40); *P* = 0.098, and non-dominant leg; ES (95%CI) = -0.31 (-0.68 to 0.07); *P* = 0.152), DLVDJ (ES (95%CI) = 0.20 (-0.17 to 0.56); *P* = 0.481) or DLFJ (ES (95%CI) = -0.49 (-0.87 to -0.10); *P* = 0.128) (Tables 2 and 3).

### Lateral trunk lean

For lateral trunk lean, within-subject differences for SLDL (dominant leg: ES (95%CI) = 0.08 (-0.29 to 0.44); *P* = 0.619; non-dominant leg: ES (95%CI) = 0.01 (-0.35 to 0.38); *P* = 0.924) and SLVDJ (dominant leg: ES (95%CI) = 0.02 (-0.34 to 0.38); *P* = 0.924; non-dominant leg; ES (95%CI) = -0.29 (-0.66 to 0.09); *P* = 0.850) were not significant (Table 2).

### Sum of knee valgus and lateral trunk lean

For the sum of knee valgus and lateral trunk lean, within-subject differences were deemed significant for SLDL (dominant leg; ES (95%CI) = 0.37 (-0.004 to 0.75); *P* = 0.033); non-dominant leg: ES (95%CI) = 0.31 (-0.07 to 0.68); *P* = 0.044), but were not significant for SLVDJ (dominant leg: ES (95%CI) = -0.32 (-0.69 to 0.05); *P* = 0.097; non-dominant leg: ES (95%CI) = -0.05 (-0.41 to 0.31); *P* = 0.860) (Table 2).

### SI

Within-subject SI differences were not significant for knee abduction (SLDL: ES (95%CI) = -0.06 (-0.42 to 0.30); *P* = 0.850; SLVDJ: ES (95%CI) = -0.61 (-1.00 to -0.21); *P* = 0.089), hip flexion (SLDL: ES (95%CI) = 0.34 (-0.03 to 0.72); *P* = 0.161; SLVDJ: ES (95%CI) = 0.13 (-0.24 to 0.49); *P* = 0.615), knee flexion (SLDL: ES (95%CI) = 0.04 (-0.32 to 0.40); *P* = 0.833; SLVDJ: ES (95%CI) = -0.20 (-0.57 to 0.17); *P* = 0.446) (Table 4).

**Table 4:** Changes of limb symmetry index (SI) during SLDL and SLVDJ for knee abduction, knee flexion and hip flexion

**Symmetry Index (%)**	**Task**	**Without KT (Baseline)**^α^	**With KT**^α^	***P value***	**Effect size**^**†**^** and 95% Confidence Interval** (Lower limit -Upper limit)	**Change Relative to Baseline**^**‡**^** (%)**
**Knee abduction**	SLDL	0.4 ± 4.8	0.2 ± 3.6	0.850	-0.06 (-0.42 to 0.30)	↓ 51.2
	SLVDJ	1.0 ± 6.5	-1.1 ± 3.5	0.089	-0.61 (-1.00 to -0.21)	↓ 213
**Knee flexion**	SLDL	7.1 ± 13.3	7.7 ± 16.6	0.833	0.04 (-0.32 to 0.40)	↑ 9.2
	SLVDJ	1.1 ± 11.0	-1.1 ± 10.7	0.446	-0.20 (-0.57 to 0.17)	↓ 200.9
**Hip flexion**	SLDL	-13.4 ± 17.0	-6.1 ± 21.1	0.161	0.34 (-0.03 to 0.72)	↑ 54.2
	SLVDJ	-6.1 ± 14.1	-3.6 ± 20.0	0.615	0.13 (-0.24 to 0.49)	↑ 41.3

Abbreviation:^α^, Values stands for Mean ± SD; *SLDL* Single-leg drop landing, *SLVDJ* Single-leg vertical drop jump, *KT* Kinesio taping; †, Cohen’s d; ‡, percent change relative to baseline (↓decrease, ↑ increase)

## Discussion

The purpose of this study was to investigate the influence of KT on trunk, hip and knee motions during a SLDL, SLVDJ, DLVDJ, and DLFJ tasks. The primary hypothesis was that KT would improve peak angles of trunk, hip and knee during SLDL, SLVDJ, DLVDJ and DLFJ. The present study results indicated that KT had an effect on the sum of knee valgus and lateral trunk lean, peak knee abduction angles during SLDL, and knee flexion during SLDL and SLVDJ tasks following a 72-h KT intervention. However, KT application showed no effect on SI during SLDL and SLVDJ tasks, or on knee and hip motions during DLVDJ or DLFJ tasks in participants with high injury risk.

As a matter of fact, peak frontal plane knee angle has been linked to high-risk biomechanics associated with ACL injury, and studies also show that knee frontal plane kinematics are coupled with hip and trunk movement [4, 17, 34, 40]. Our results showed that peak knee abduction angle was reduced only in SLDL after KT application. This finding is consistent with a study conducted by Rajasekar et al. [35], who reported that KT on the gluteus medius improved activation, and reduced knee abduction angle during a double-leg drop jump test after a 72-h intervention. Indeed, using KT improves motor unit recruitment [2, 31]. Therefore, KT might be added as an adjunct along with routine muscle strengthening to favourably modulate high-risk movement. After 72 h, moreover, KT caused the sum of knee valgus and lateral trunk lean to be increased during SLDL, possibly by changes in knee abduction angle. The percentage changes to the baseline showed that after 72 h KT the sum of knee valgus and lateral trunk lean and knee abduction angles decreased in the SLDL. These factors can reduce potential knee injury.

The basic KT mechanisms have not heretofore been fully investigated. It has been claimed that KT improves blood circulation and lymphatic flow, neurological activation, corrects weak muscle function, and enhances joint function [15, 20, 29, 31, 43, 45]. As KT stretches the skin, it stimulates cutaneous mechanoreceptors, which may cause physiological changes in the area and affect knee flexion angle [15, 20, 29, 31, 43, 45]. KT effects on muscle stretching appear after a relatively long time [31]. The longer use of KT application (72 h) possibly generated higher chronic stimulation of skin mechanoreceptors [31, 32]. Rebolledo et al. [31] revealed that KT may improve jump performance after 72 h. However, it should not be overlooked that their study evaluated the jump height of a countermovement jump and squat jump, whereas the current study investigated a series of drop landings.

The results obtained from this study demonstrated that KT did not affect knee and hip angles during TLDVJ and DLFJ tasks. In fact, these results contradicted those observed in studies conducted by Limroongreungrat et al. [26], who showed that using ACL-KT technique can change DVJ task patterns in healthy participants. They reported that using the ACL-KT technique with a 75% tension reduces knee abduction angle during a DVJ. Furthermore, KT provides tactile stimulation [23, 44], although KT tactile inputs may not be strong enough to modulate muscle strength [13], and consequently, unable to moderate joint angles in healthy athletes during the double-leg landings of TLDVJ and DLFJ.

Decreased maximal flexion of the knee and hip are risk factors associated with ACL injury. Therefore, increasing knee and hip flexion during jump landing are components of successful injury prevention programs [25]. The present study showed that using KT increases knee flexion in SLDL and SLVDJ, which results in less strain within the ACL [14]. Similarly, Pelletier et al. [34] reported that KT tape increases knee and hip flexion angle during running, serving to better absorb impact forces. This finding may be explained by two theories. One theory is that KT results in increasing blood circulation to the taped area, thus affecting muscle and myofascial function and physiology. The other theory implies that the cutaneous mechanoreceptors are stimulated by KT, and this stimulation may affect the joint angle [45]. The main mechanism whereby KT accomplishes these aims is purportedly the stimulation of cutaneous mechanoreceptors, thereby improving proprioception and joint position sense [26]. The possible increase in blood circulation [45], and eccentric hamstring muscle control affects knee flexion angle [11]. With KT applied on the quadriceps and hamstring muscles, greater knee flexion is facilitated, which then has a positive effect on hamstring function [2, 31], This factor can theoretically be useful and effective for individuals with high ACL injury risk, who often perform difficult landings and have minimal sagittal plane knee displacement.

In contrary to the hypothesis, the results in this study indicate that KT does not affect maximal hip flexion in any landing test. Although no significant statistical increase was observed for maximum hip flexion angle during SLDL and SLVDJ, there was a small EF for dominant limb (ES = -0.53), trivial EF for non-dominant limb (ES = -0.34) during SLDL and moderate EF for dominant limb (ES = -0.82), and trivial EF for non-dominant limb (ES = -0.31) during SLVDJ. In fact, this finding is different from the results reported by Pelletier et al. [34], a difference that might be attributable to task type, participant gender, or measurement tools. This factor may be related to the nature of KT technique, which is designed to imitate human skin. It does not stabilize the tissue, but permits full range-of-motion. Over-stretching of the KT may have hindered our ability to detect a statistically significant differences in hip flexion angles and lateral trunk lean between the KT conditions.

In addition, this study showed that the extent upon which KT affects SI during SLDL and SLVDJ was not statistically significant. The cause may be attributed to the equal effect of the KT on dominant and non-dominant legs. To the authors’ knowledge, no study has explored SI after KT application during landing. Hence, it is not possible to compare the present results with previous findings. However, various studies indicate that KT increases motor unit recruitment in healthy individuals, which might justify biomechanical changes affected by the KT technique [2, 31, 44]. Furthermore, the results revealed that KT has no effect on lateral trunk lean angle. This finding is consistent with Yoshida et al. [45] who reported that lateral trunk lean is not affected by KT.

As in all studies, limitations have arisen that might affect the results. Specifically, this study population did not include a sham/placebo group. 2D motion analysis has been used to evaluate lower extremity kinematics during functional tasks in healthy and injured individuals. However, it is not without its flaws. Further studies through which electromyography and three-dimensional (3D) motion analysis are used to detect changes in muscle excitability and joint kinematics are warranted. One of the weaknesses in this study is the fact that female participants were excluded. Future clinical trials with a female group and larger sample size should be performed to investigate the effect of KT on joint kinematics. Future studies are needed to assess the follow-up and long-term effects of KT to have strong conclusions.

## Conclusions

In conclusion, a 72-h KT application may improve peak knee abduction angle, sum of knee valgus and lateral trunk motion during SLDL, and knee flexion during SLDL and SLVDJ. However, KT resulted in no significant effect on knee and hip joint angles during TLDVJ and DLFJ tasks, nor SI during SLDL and SLVDJ.

## Abbreviations

ACL: Anterior Cruciate Ligament
KV: Knee Valgus
2D: Two-Dimensional
SLVDJ: Single Leg Vertical Drop Jump
GRF: Ground Reaction Force
KT: Kinesio Taping
SLDL: Single-Leg Drop Landing
DLVDJ: Double leg Vertical Drop Jump
DLFJ: Double Leg Forward Jump
SLS: Single Leg Squat Test
AC: Acromioclavicular
ASIS: Anterior Superior Iliac Spine
GT: Greater Trochanter
SI: Symmetry Index
ES: Effect Size
3D: Three-Dimensional
